# Acute Kidney Injury After Endoscopic Ureterocele Incision in a Duplex System with Contralateral Multicystic Dysplastic Kidney: From Obstructive Complication to Surgical Resolution—A Case Report

**DOI:** 10.3390/reports9030251

**Published:** 2026-08-03

**Authors:** Konstantinos Gkialas, Anna Papakonstantinou, Evangelos Fragkiadis, Napoleon Moulavasilis, Panagiotis Mitsos

**Affiliations:** 1 Second Department of Urology, Sismanogleio Hospital, School of Medicine, National and Kapodistrian University of Athens, 15126 Athens, Greece; k.gkialas@gmail.com; 2 Department of Urology, General Children’s Hospital “Agia Sofia”, Thivon 1 & Papadiamantopoulou, 11527 Athens, Greece; mitsospanagiotis@yahoo.com; 3 First Department of Urology, Laiko Hospital, School of Medicine, National and Kapodistrian University of Athens, 11527 Athens, Greece; e.fragkiadis@gmail.com (E.F.); napomoul@hotmail.com (N.M.)

**Keywords:** ureterocele, duplex collecting system, multicystic dysplastic kidney, acute kidney injury, nephrostomy, pediatric urology, hydronephrosis, metabolic acidosis

## Abstract

**Background and Clinical Significance**: Endoscopic ureterocele incision is the preferred initial treatment for ureteroceles associated with duplex collecting systems due to its capability for rapid decompression via a minimally invasive technique with generally favorable outcomes among pediatric patients. However, the postoperative trajectory in children with solitary functioning renal units remains inadequately characterized. We present a severe, yet reversible, case of postrenal acute kidney injury (AKI) following endoscopic ureterocele incision in an infant with a contralateral multicystic dysplastic kidney (MCDK). This case emphasizes the pathophysiological implications of failed ureterocele decompression and the vital importance of rigorous postoperative monitoring. **Case Presentation**: A female infant with a right MCDK and a left duplex collecting system featuring an upper pole ureterocele underwent transurethral endoscopic incision due to progressive hydronephrosis. Within 24 h following surgery, the patient exhibited oliguria, oedema, worsening hydronephrosis, hyponatremia (125 mmol/L), metabolic acidosis, and increasing serum creatinine levels, indicative of postrenal AKI. Arterial blood gas analyses indicated severe renal-driven metabolic acidosis with bicarbonate levels of 13.9 mmol/L, accompanied by respiratory compensation and normal lactate levels. Imaging studies revealed deteriorating hydronephrosis of the upper and lower poles of the left kidney. Emergency open nephrostomy placement in the lower pole, after failed jj insertion in the lower pole ureteral orifice, resulted in the immediate restoration of urinary drainage and progressive biochemical recovery. The patient required a brief period of intensive care monitoring, followed by hospitalization in pediatric and urological departments. Longitudinal imaging demonstrated persistent but stable upper pole dilatation with preserved parenchyma. Definitive management was later achieved through right nephrectomy of the non-functioning MCDK. **Conclusions**: In patients with solitary functioning renal units, the endoscopic ureterocele incision may result in postoperative local oedema, potentially leading to clinically significant obstructive AKI. This case underscores the necessity for intensified surveillance and individualized postoperative management strategies in anatomically complex pediatric patients.

## 1. Introduction and Clinical Significance

Congenital anomalies of the kidney and urinary tract (CAKUT) are among the leading causes of chronic kidney disease in pediatric populations worldwide [1]. Within this spectrum of abnormalities, ureteroceles are recognized as a relatively uncommon but clinically significant condition, particularly when associated with duplex collecting systems [2]. A ureterocele is characterized by cystic dilatation of the distal intravesical ureter, stemming from congenital stenosis of the ureteral orifice and incomplete canalization of the Chwalla membrane during embryonic development [2]. Although ureteroceles can occur in single collecting systems, they are most frequently associated with duplex systems in pediatric patients, especially in female infants [3].

The clinical manifestations of ureteroceles can vary widely based on anatomical configuration, degree of obstruction, associated vesicoureteral reflux, and residual renal function [3]. Prenatal ultrasonography has meaningfully facilitated the timely identification of hydronephrosis and related upper urinary tract abnormalities during fetal development [4]. Nevertheless, the postnatal progression remains unpredictable. While some children may remain asymptomatic, others may develop recurrent urinary tract infections, obstructive hydronephrosis, bladder outlet obstruction, voiding dysfunction, or progressive deterioration of renal function [2,3].

Management strategies for ureteroceles remain a topic of ongoing debate and continue to evolve. According to the European Association of Urology (EAU) and European Society for Paediatric Urology (ESPU) guidelines, timely drainage is essential for managing obstructing lesions [2]. Historically, open reconstructive surgery was the standard therapeutic approach; however, advancements in pediatric endourology have led to a paradigm shift toward minimally invasive decompression techniques [3]. Transurethral endoscopic incision or puncture of the ureterocele is now widely accepted as the first-line treatment in numerous medical centers, as it allows for rapid decompression, minimizes the need for major surgery during infancy, and may help preserve renal function while facilitating future reconstruction if necessary [2,5].

Despite its widespread acceptance, the postoperative course following endoscopic ureterocele incision is not uniformly benign. Reported complications include persistent or recurrent obstruction, recurrent hydronephrosis, vesicoureteral reflux, urinary tract infections, the need for repeat endoscopic intervention, and secondary reconstructive procedures [5]. Reported outcomes can vary significantly, particularly in patients with ectopic ureteroceles, duplex systems, severe upper pole dysplasia, or diminished contralateral renal reserve [3,5].

Children with a MCDK and contralateral obstructive anomalies represent a particularly susceptible subgroup [6]. MCDK is one of the most prevalent congenital renal abnormalities, characterized by non-functioning renal parenchyma replaced by multiple non-communicating cysts and dysplastic tissue. In cases of unilateral disease, prognosis is generally favorable, provided that the contralateral kidney is functioning normally [6]. However, the coexistence of contralateral obstructive uropathy can transform these patients into carriers of a functional solitary kidney, making them highly vulnerable to any reduction in urinary drainage or renal perfusion [6,7].

AKI resulting from failed decompression after endoscopic ureterocele incision is underrepresented in the medical literature. Most reports focus on persistent hydronephrosis, reflux, or the necessity for secondary surgery, rather than on severe metabolic deterioration that requires emergency urinary diversion. Additionally, few studies specifically address postoperative biochemical evolution and acid-base disturbances in infants with solitary functioning renal units [7].

This report details a severe but reversible episode of postrenal AKI that occurred following transurethral endoscopic ureterocele incision in an infant with an ipsilateral duplex collecting system and contralateral MCDK. This case illustrates the pathophysiological consequences of failed decompression in a solitary functioning renal unit, the rapid metabolic decline that may follow acute obstruction, and the essential nature of immediate urinary diversion and intensive postoperative surveillance.

## 2. Case Presentation

A female infant with prenatally detected hydronephrosis underwent postnatal evaluation after antenatal ultrasonography demonstrated marked left-sided urinary tract dilatation and suspected a congenital abnormality of the right kidney. Upon conducting a thorough postnatal ultrasonographic examination, the findings confirmed the presence of a right multicystic kidney, with numerous cortical cysts of varying sizes, a notable loss of corticomedullary differentiation, increased echogenicity, and minimal identifiable functional renal parenchyma. In contrast, the left kidney exhibited a duplex collecting system that showed marked dilatation of the upper pole moiety, accompanied by associated ureteral dilatation extending distally toward the bladder. Additionally, a cystic intravesical lesion consistent with a ureterocele was identified at the left ureterovesical junction. Remarkably, the upper pole moiety preserved satisfactory cortical thickness and demonstrated normal corticomedullary differentiation, suggesting adequate function. To further evaluate renal function, subsequent renal cortical scintigraphy was performed using technetium-99m dimercaptosuccinic acid (Tc-99m DMSA). This imaging study revealed that the left kidney accounted for approximately 92% of the total renal function, whereas the right kidney contributed a mere 8%, thereby confirming the presence of a functionally solitary renal unit. Throughout infancy, serial ultrasonographic examinations revealed progressive upper pole hydronephrosis affecting the left duplex system. Serum creatinine levels remained within the expected physiological range for her age during the first two months of life, without evidence of a progressive upward trend. The progressive ureteral dilatation and persistent upper moiety obstruction ultimately led the medical team to decide on surgical intervention for decompression. There was no known family history of congenital renal or urinary tract anomalies, other congenital malformations, or similarly affected siblings, suggesting a sporadic presentation.

In November 2024, the infant (aged approximately 2 months old) underwent a transurethral endoscopic incision of the ureterocele, conducted under general anesthesia. During the procedure, endoscopic visualization confirmed the presence of a large intravesical ureterocele originating from the upper pole ureter of the duplex system and a normal ureteral orifice of the lower moiety of the left kidney. A transurethral incision with cautery was performed to facilitate decompression of the ureterocele. The surgical intervention was completed without any immediate complications, and the initial postoperative urine output was deemed satisfactory. Following the procedure, the patient was promptly transferred to the Clinic for postoperative observation.

**Figure 1:**
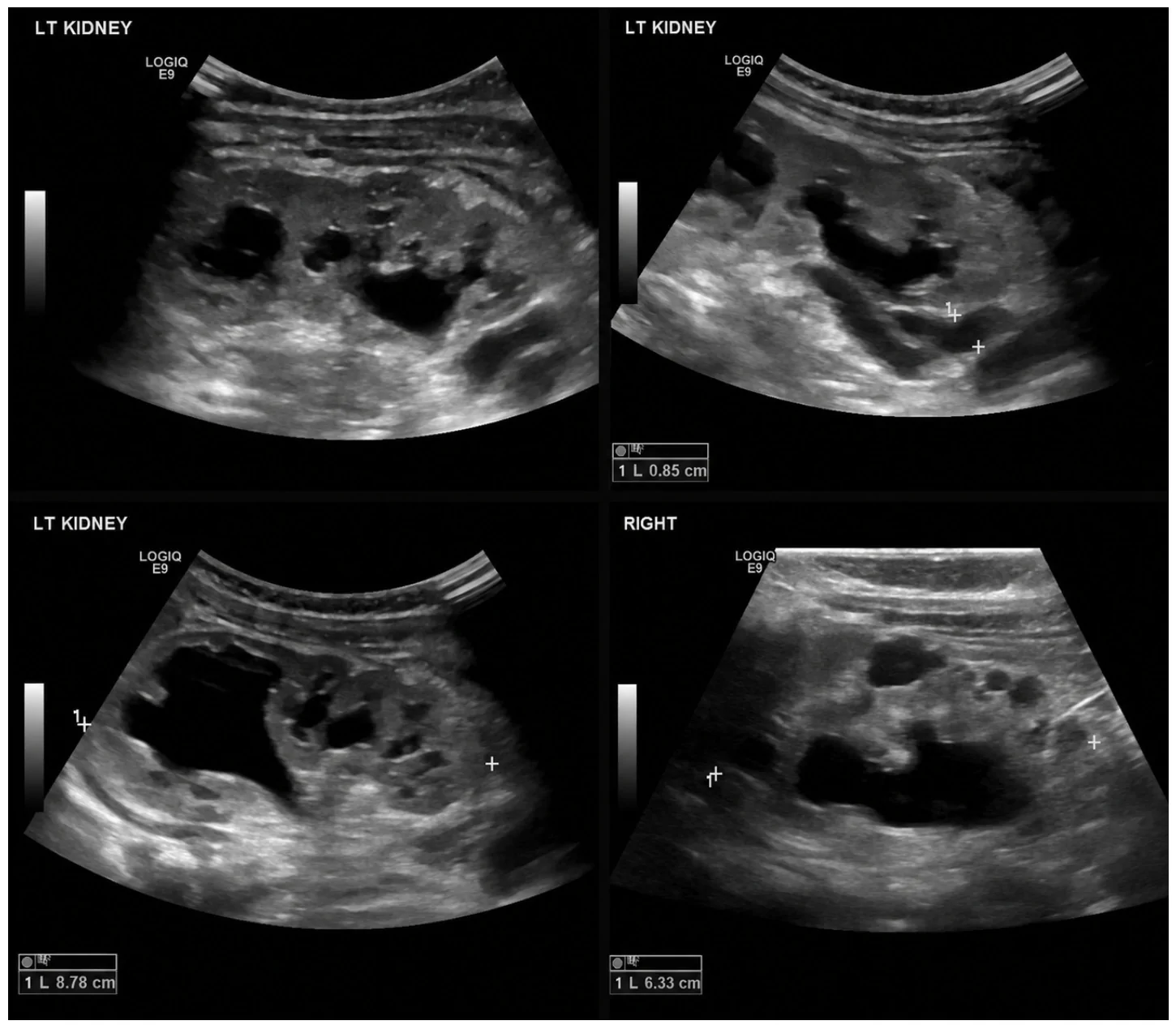
Post-op renalultrasound demonstrating worsening hydronephrosis of both the upper- and lower-pole moieties of the left duplex collecting system, with persistent ureteral dilatation. The contralateral right MCDK is also shown.

**Figure 2:**
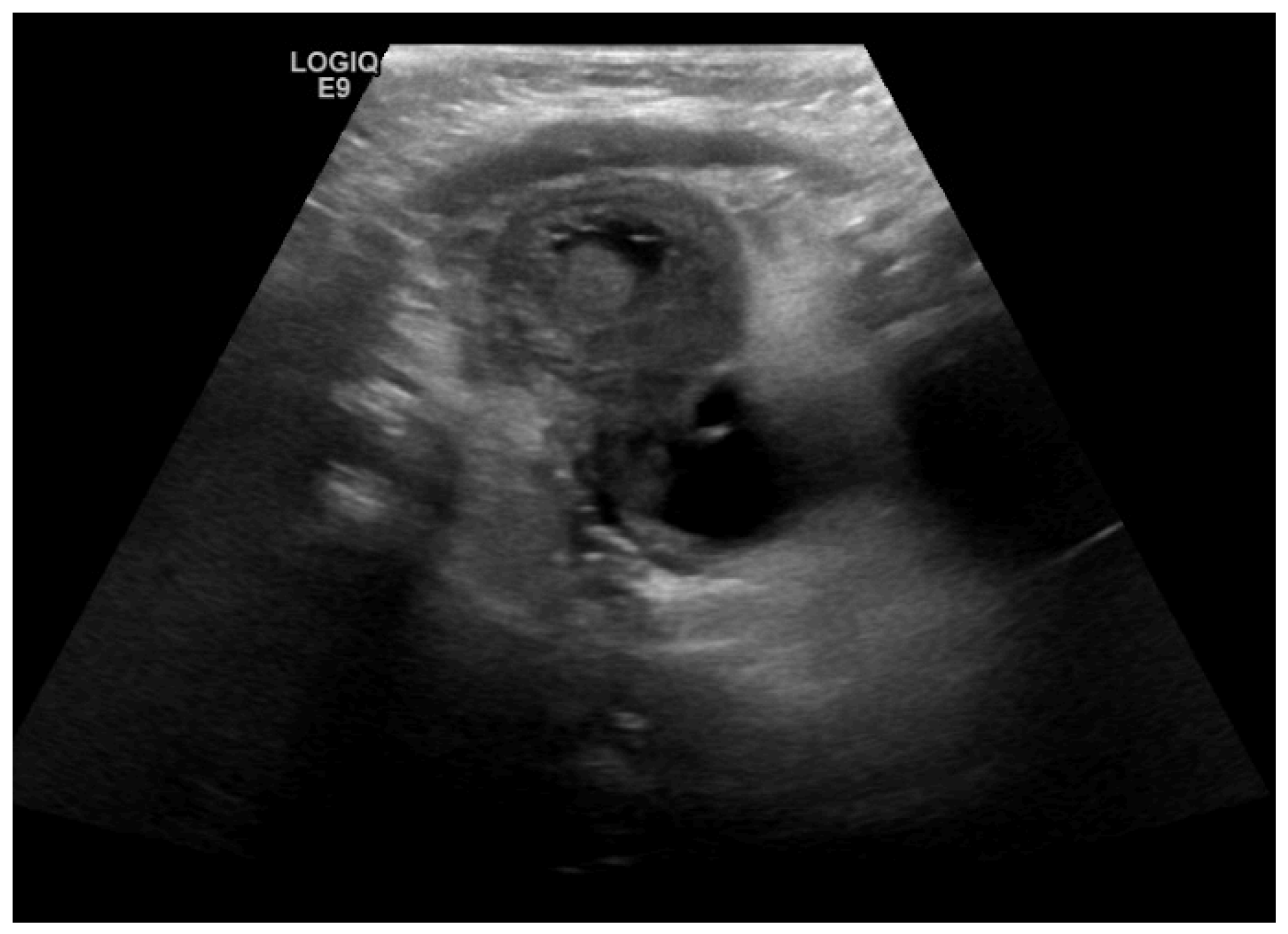
Pre-nephrostomy bladder ultrasound obtained during the acute postoperative deterioration.

Within 24 h after surgery, the patient developed progressive oliguria, worsening oedema, and clinical deterioration. Laboratory investigations revealed a troubling rise in serum creatinine levels (1.38 mg/dL), along with significant electrolyte abnormalities, particularly hyponatremia, with sodium levels plummeting to 125 mmol/L. A follow-up ultrasound of the kidneys and bladder showed worsening hydronephrosis of the upper and lower moieties of the left kidney. Both the upper pole collecting system and the associated ureter remained markedly dilated, with echogenic material within the dilated collecting system, likely representing hemorrhagic debris or urinary stasis secondary to acute obstruction. The lower pole also showed ureterohydronephrosis, suggesting obstruction of the lower moiety as well (Figure 1 and Figure 2). Arterial blood gas analyses further demonstrated significant metabolic acidosis. Serial measurements revealed pH values fluctuating between 7.37 and 7.39, with bicarbonate concentrations dropping as low as 13.9 mmol/L, and base excess values reaching −9 mmol/L. At the same time, the partial pressure of carbon dioxide ranged between 23 and 27 mmHg, indicating appropriate respiratory compensation. Importantly, lactate levels remained within normal limits throughout this episode, effectively ruling out systemic hypoperfusion or sepsis as potential causes of the acid-base disturbance. The combination of oliguria, worsening hydronephrosis, electrolyte imbalances, metabolic acidosis, and rising creatinine levels established a diagnosis of obstructive postrenal AKI.

Given the rapid progression of renal dysfunction in the context of a solitary functioning kidney, emergency urinary diversion became imperative. On post-op day 1, we performed a cystoscopy and initially tried to catheterize the left lower pole orifice; however, we were unsuccessful due to extensive oedema. Subsequently, we performed an emergency open nephrostomy placement to the lower pole of the left kidney. Following the nephrostomy insertion, immediate restoration of urine output was observed, with progressive improvement in renal function and metabolic parameters over the subsequent hours. 

Because of severe metabolic derangement, the patient was admitted to the ICU for close monitoring, fluid management, electrolyte correction, and serial laboratory assessment. During her stay in the ICU, meticulous fluid management, electrolyte correction, and serial laboratory evaluations were conducted to ensure her stability. Follow-up arterial blood gas analyses obtained the next day demonstrated significant improvement. The pH normalized to 7.385, bicarbonate levels increased to 19.7 mmol/L, and the base excess improved to −4.7 mmol/L. The partial pressure of carbon dioxide was recorded at 33.7 mmHg, consistent with ongoing but improving respiratory compensation. Sodium levels returned to normal at 136 mmol/L, while serum creatinine progressively declined (0.55 mg/dL 10 h later, 0.32 mg/dL 24 h later). Crucially, lactate levels remained within normal limits throughout the postoperative course, supporting the conclusion that the metabolic deterioration was primarily attributed to acute obstructive renal dysfunction rather than systemic circulatory compromise.

After 24 h of intensive care monitoring and stabilization, the patient was successfully transferred to the pediatric ward, where she remained hospitalized for an additional four days under conservative management. Clinical examinations demonstrated a progressive recovery of urine output, resolution of fluid imbalance, and normalization of laboratory parameters. Following this, the patient continued her hospitalization in the pediatric urology department for an additional five days. Nephrostomy drainage remained satisfactory, and no infectious complications were documented throughout her stay.

**Figure 3:**
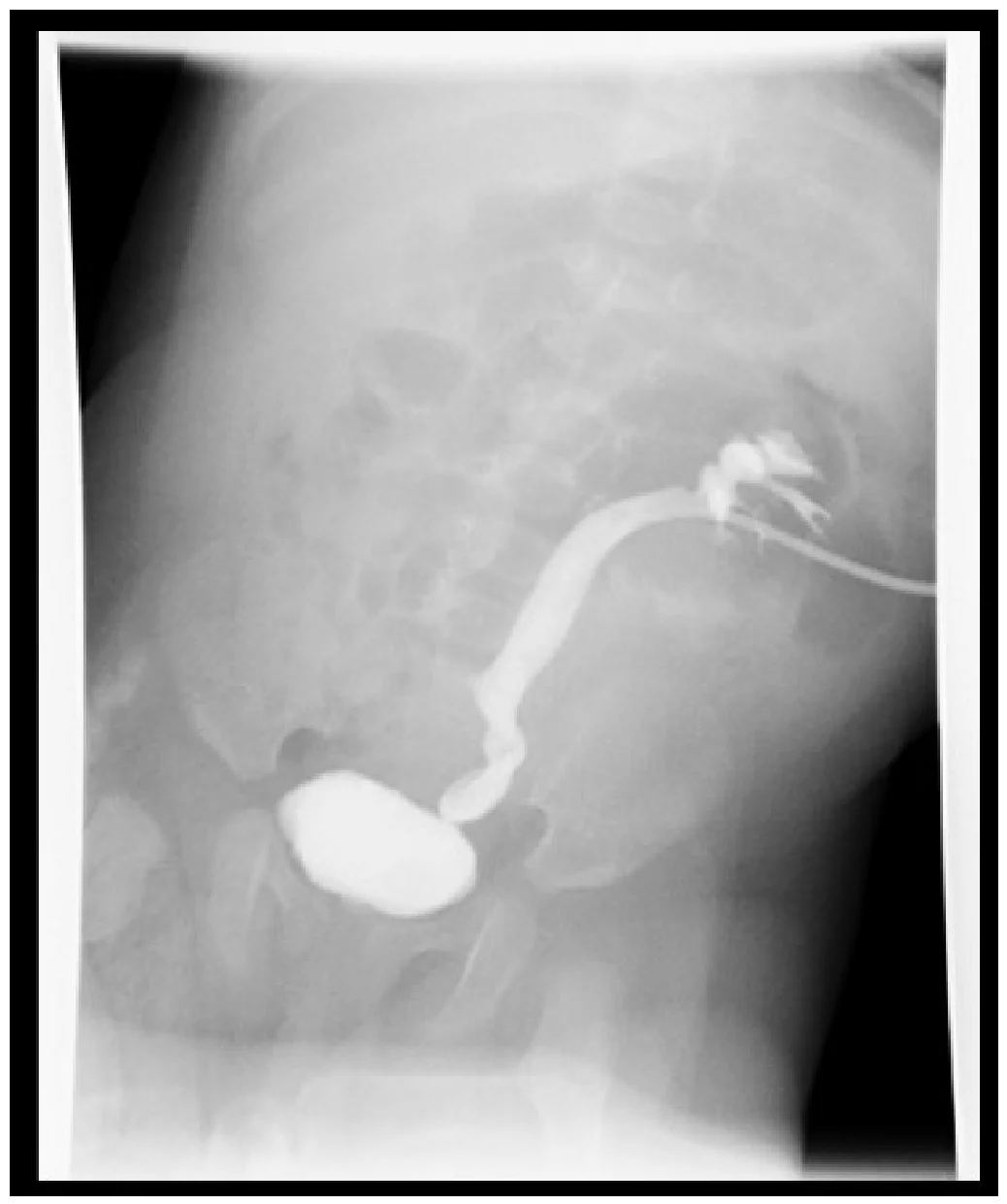
Nephrostomography performed four weeksafter the opennephrostomy placement demonstrating unobstructed contrast passage, confirming restoration of urinary drainage and resolution of the postoperative obstruction.

Approximately 4 weeks later, we performed a nephrostomography to check that the urinary drainage had been ultimately restored (Figure 3). The nephrostomy tube was subsequently removed. However, due to persistent residual metabolic acidosis despite renal function recovery, oral bicarbonate supplementation was initiated and continued during nephrological outpatient follow-up. This treatment was gradually tapered and eventually discontinued 6 months later, following sustained normalization of acid-base balance and biochemical parameters.

Longitudinal ultrasonographic follow-up revealed a dynamic yet overall stable postoperative evolution. The right kidney consistently displayed the characteristics of a MCDK, including multiple cortical cysts, significant parenchymal loss, and no evidence of functional recovery. The left kidney with the duplex collecting system continued to exhibit persistent but stable dilatation of the upper pole moiety observed throughout follow-up. Mild residual ureteral dilatation was also noted, while cortical thinning of the upper pole remained stable without signs of progressive deterioration. Importantly, the lower pole moiety preserved satisfactory cortical thickness and maintained a normal sonographic appearance. 

**Figure 4:**
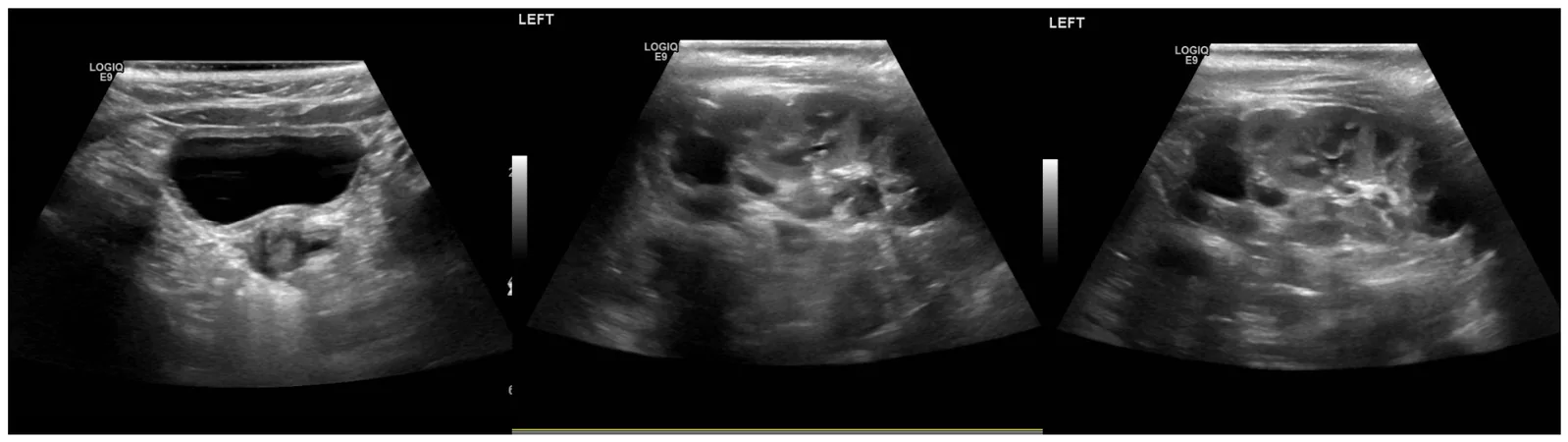
Follow-up renal ultrasound obtained one month after nephrostomy removal, demonstrating persistent but stable upper-pole dilatation, resolution of the ureterocele, preserved lower-pole parenchyma, and no evidence of recurrent obstruction.

**Figure 5:**
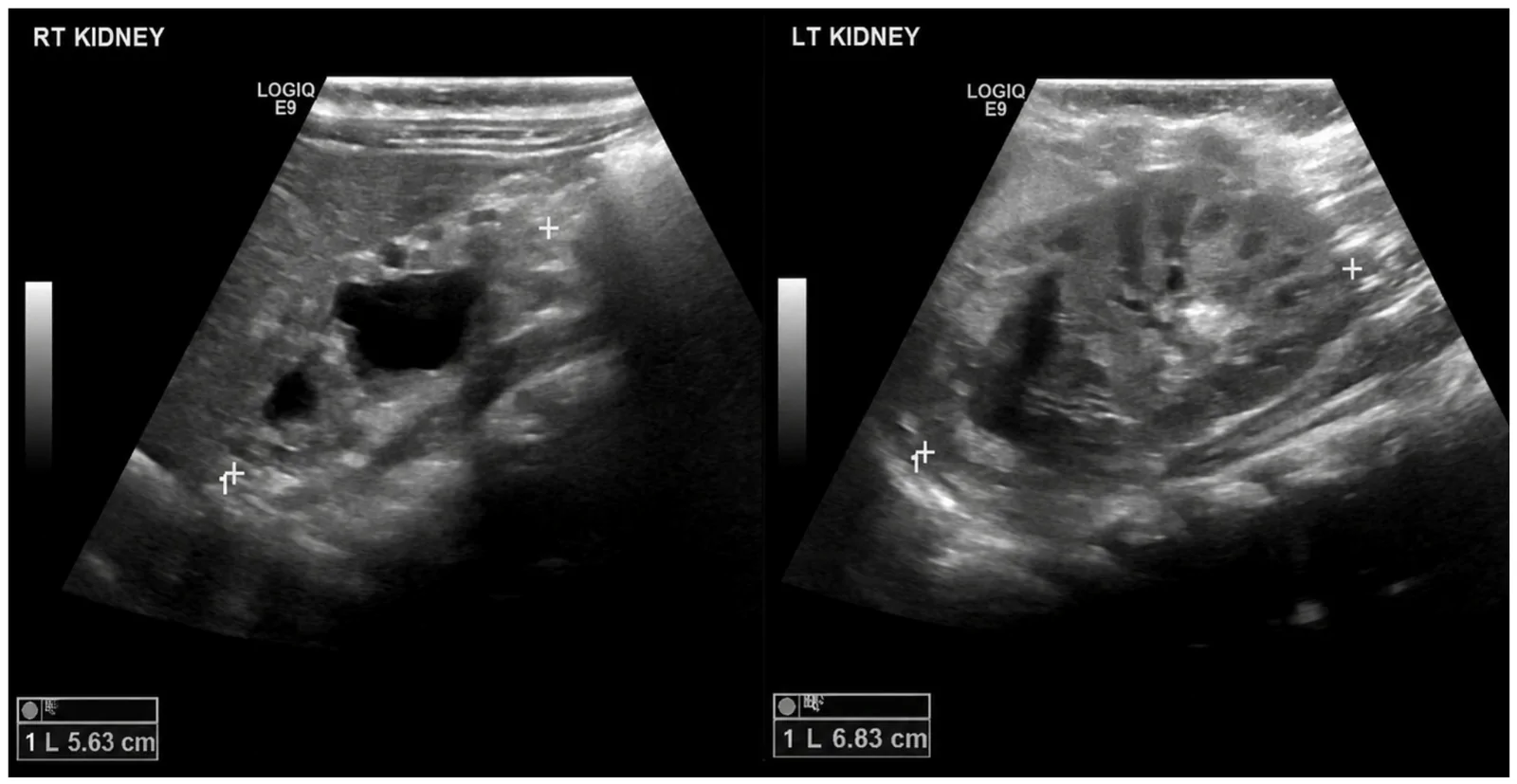
Follow-up renal ultrasound four months after nephrostomy removal demonstrating stable postoperative findings. Persistent size and multicystic dysplastic morphology of the right kidney.

Long-term follow-up confirmed stable function of the left kidney, with no further episodes of renal deterioration (Figure 4 and Figure 5). The endoscopic ureterocele incision proved effective, with no evidence of recurrent obstruction during follow-up. Given the persistent size and multicystic dysplastic morphology of the right kidney, together with its minimal contribution to differential renal function (8% on Tc-99m DMSA), right nephrectomy was performed one year later after continued follow-up. The postoperative recovery was uneventful, and follow-up evaluations demonstrated preserved left renal function, satisfactory urinary drainage, and the absence of recurrent metabolic abnormalities. This comprehensive management approach effectively addressed the complexities associated with the patient’s renal anomalies and ensured her long-term well-being.

To improve clarity, the chronological sequence of postoperative events, including clinical deterioration, laboratory and imaging findings, therapeutic interventions, and long-term follow-up, is summarized in Table 1.

## 3. Discussion

Endoscopic ureterocele incision has become one of the most commonly utilized minimally invasive procedures in pediatric urology, as it provides rapid decompression with limited surgical morbidity and may postpone or even eliminate the need for major reconstructive surgery during infancy [2]. Nevertheless, postoperative outcomes can vary widely, particularly in patients with duplex systems and ectopic ureteroceles. Recent studies have shown that duplex systems and ectopic ureteroceles are significantly associated with higher rates of secondary interventions following endoscopic decompression [3,5]. This underscores the limitations of standard minimally invasive strategies in anatomically complex pediatric patients and suggests the need for tailored postoperative surveillance protocols.

In the present case, the coexistence of a contralateral MCDK considerably reduced the functional renal reserve. Consequently, transient postoperative obstruction quickly led to clinically relevant AKI. Children with solitary functioning kidneys possess limited compensatory capacity, making them more vulnerable to acute postoperative deterioration [6,7].

The precise mechanism behind failed decompression remains uncertain. A transient inflammatory response at the site of ureterocele incision is the most likely explanation. Postoperative oedema at the incision site may temporarily impair urinary drainage, while the collapse of the decompressed ureterocele or ongoing ureteral dysmotility might contribute to functional obstruction despite technically adequate incision [2]. Although the upper- and lower-pole ureteral orifices are anatomically distinct in duplex collecting systems, a large intravesical ureterocele may distort the trigonal anatomy and alter their spatial relationship. In this setting, postoperative inflammatory oedema extending beyond the incision site, possibly exacerbated by thermal injury from cautery, could transiently impair drainage from both moieties. In our patient, postoperative ultrasound demonstrated worsening hydronephrosis of both renal moieties together with echogenic material within the collecting system, supporting the hypothesis of transient obstruction affecting both ureteral drainage pathways.

The metabolic abnormalities observed during the deterioration were consistent with acute obstructive renal dysfunction. Arterial blood gas analysis demonstrated metabolic acidosis with appropriate respiratory compensation, and persistently normal lactate levels indicated a renal origin rather than systemic hypoperfusion or sepsis. Hyponatremia likely reflected impaired free water excretion and altered tubular sodium handling during the acute obstruction, a phenomenon documented in pediatric AKI [8,9].

Renal function improved rapidly following open nephrostomy placement. The consensus by the EAU/ESPU guidelines issues a strong recommendation to offer immediate drainage, such as endoscopic decompression or percutaneous nephrostomy, for patients presenting with obstructive uropathy or failed decompression [2]. The temporal relationship between decompression and biochemical recovery strongly supports the diagnosis of reversible obstructive AKI. In pediatric obstructive uropathy cases, prompt urinary diversion is essential when renal function is under threat [2,10].

Most ureterocele series assess heterogeneous populations and rarely focus specifically on solitary functioning renal units. As a result, the incidence of severe postoperative AKI in this subgroup remains poorly defined [11]. This case suggests that routine postoperative observation may be inadequate for selected high-risk patients. Early postoperative imaging, serial biochemical reassessment, and stringent monitoring of urine output may facilitate the recognition of evolving obstruction before irreversible renal injury occurs.

Long-term follow-up showed that persistent mild upper pole dilatation during follow-up did not correlate with progressive deterioration of the lower pole moiety or overall renal function. The stable preservation of lower pole parenchyma supports the strategy of conservative surveillance following recovery.

Persistent upper pole dilatation following endoscopic decompression does not necessarily mandate further intervention. Nevertheless, children with duplex collecting systems require long-term surveillance, as secondary reconstructive procedures—including ureteroureterostomy, ureteral reimplantation, or upper pole heminephrectomy—may become necessary in cases of recurrent obstruction, vesicoureteral reflux, recurrent urinary tract infections, or progressive deterioration of upper moiety function. Although the upper pole remained mildly dilated throughout follow-up in our patient, no clinical or radiological evidence of recurrent obstruction, urinary tract infection, or deterioration of renal function was observed. Therefore, conservative surveillance was considered appropriate, while recognizing that future reconstructive intervention may still become necessary should these complications develop. 

Although a direct embryological relationship between MCDK and duplex collecting system with ureterocele has not been conclusively demonstrated, these anomalies may represent different phenotypic manifestations within the broader spectrum of CAKUT [12,13].

## 4. Conclusions

Endoscopic ureterocele incision is an effective and minimally invasive treatment for managing duplex systems associated with ureterocele [2,3]. However, in patients with solitary functioning renal units, postoperative obstruction may occur despite technically successful interventions, potentially leading to clinically significant AKI. The current case underscores the potentially severe metabolic consequences of failed decompression in infants with limited renal reserve.

Early recognition of postoperative oliguria, worsening hydronephrosis, electrolyte abnormalities, and metabolic acidosis are critical for timely intervention. Prompt urinary diversion through emergency nephrostomy, aligned with current international guidelines, enabled rapid reversal of renal dysfunction and preservation of long-term renal function in this patient. These findings highlight the importance of intensified postoperative surveillance and individualized management strategies in pediatric patients with complex urinary tract anatomy and solitary functioning kidneys.

## Figures and Tables

**Table 1:** Chronological timeline of the patient’s postoperative course.

Time	Clinical Event	Key Findings/Intervention
November 2024 (Age: ~2 months)	Endoscopic ureterocele incision	Transurethral incision performed without intraoperative complications. Initial postoperative urine output satisfactory.
Within 24 h	Clinical deterioration	Progressive oliguria, generalized oedema, worsening hydronephrosis on ultrasound. Serum creatinine increased to 1.38 mg/dL, sodium 125 mmol/L, bicarbonate 13.9 mmol/L, metabolic acidosis with normal lactate.
	Repeat ultrasound	Marked worsening hydronephrosis involving both upper and lower moieties with persistent ureteral dilatation and echogenic intraluminal material suggestive of obstruction.
Postoperative Day 1 after Transurethral incision	Emergency intervention	Cystoscopy: catheterization of the lower pole ureter unsuccessful due to severe oedema. Emergency open nephrostomy placement.
Postoperative Day 1 after nephrostomy	ICU admission	Intensive monitoring, fluid and electrolyte management. Serum creatinine decreased to 0.55 mg/dL (10 h) and 0.32 mg/dL (24 h). Sodium normalized (136 mmol/L). Bicarbonate improved to 19.7 mmol/L.
	Clinical improvement	Prompt restoration of urine output with progressive biochemical recovery.
Post-nephrostomy Days 2–10	Inpatient recovery	4 days in the pediatric ward and 5 days in the pediatric urology department. Stable nephrostomy drainage without infectious complications.
4 weeks postoperatively	Nephrostomography	Confirmed unobstructed urinary drainage. Nephrostomy tube removed.
	Medical treatment	Oral bicarbonate supplementation initiated because of persistent residual metabolic acidosis despite recovery of renal function.
6 months postoperatively	Follow-up	Bicarbonate therapy gradually discontinued after sustained normalization of acid-base status and biochemical parameters.
1 year postoperatively	Definitive surgery	Right nephrectomy for the non-functioning MCDK. Uneventful recovery with preserved function of the solitary left kidney.

## Data Availability

The original contributions presented in this study are included in the article. Further inquiries can be directed to the corresponding author.

## Abbreviations

The following abbreviations are used in this manuscript:

AKI	Acute kidney injury
MCDK	Multicystic dysplastic kidney
CAKUT	Congenital anomalies of the kidney and urinary tract
EAU	European Association of Urology
ESPU	European Society for Paediatric Urology
Tc-99m DMSA	Technetium-99m dimercaptosuccinic acid
